# Supplementary material for: Rethinking Protein Drug Design with Highly Accurate Structure Prediction of Anti-CRISPR Proteins
Source: Pharmaceuticals (Basel). 2022 Mar 4;15(3):310. doi: 10.3390/ph15030310 (PMC8949011; doi:10.3390/ph15030310)
Supplement: Supplementary file 1 [file pharmaceuticals-15-00310-s001.zip › pharmaceuticals-1593642-supplementary.pdf]

Supplementary Materials

Rethinking Protein Drug Design with Highly Accurate Structure Prediction of Anti-CRISPR Proteins

Ho-Min Park [1,2], Yunseol Park [1], Joris Vankerschaver [1,3], Arnout Van Messem [4], Wesley De Neve [1,2] and Hyunjin Shim [1,*]

[1] Center for Biosystems and Biotech Data Science, Ghent University Global Campus, Incheon 21985, Korea; homin.park@ghent.ac.kr (H.-M.P.); yunseol.park@ghent.ac.kr (Y.P.); joris.vankerschaver@ghent.ac.kr (J.V.); wesley.deneve@ghent.ac.kr (W.D.N.)
[2] Department of Electronics and Information Systems, Ghent University, B-9000 Ghent, Belgium
[3] Department of Applied Mathematics, Computer Science and Statistics, Ghent University, B-9000 Ghent, Belgium
[4] Department of Mathematics, University of Liège, 4000 Liège, Belgium; arnout.vanmessem@uliege.be
* Correspondence: hyunjin.shim@ghent.ac.kr

Figure S1. The performance of AlphaFold2 on the Acr protein datasets in comparison to the CASP14 dataset using RMSD. The closer the TM-score is to 0, the more similar the predicted structure is to its true experimental structure. (Set A: Verified Acr proteins with experimental structures, Set C: Putative Acr proteins with experimental structures).

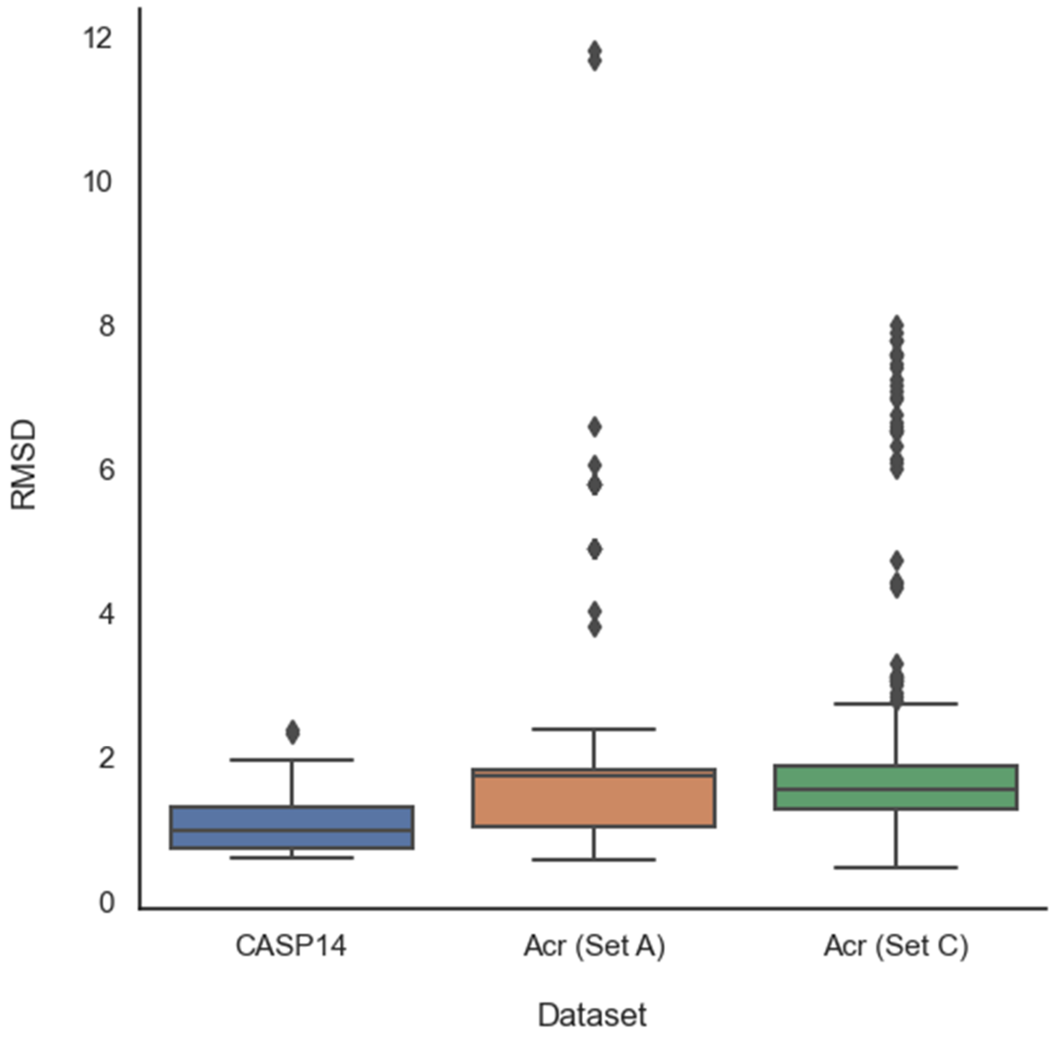

Figure S2. The structural tree of anti-CRISPR proteins reconstructed using structure-based methods (Set A + Set B; *n* = 207). The AlphaFold-predicted protein structures illustrate representative structural forms for each clade.

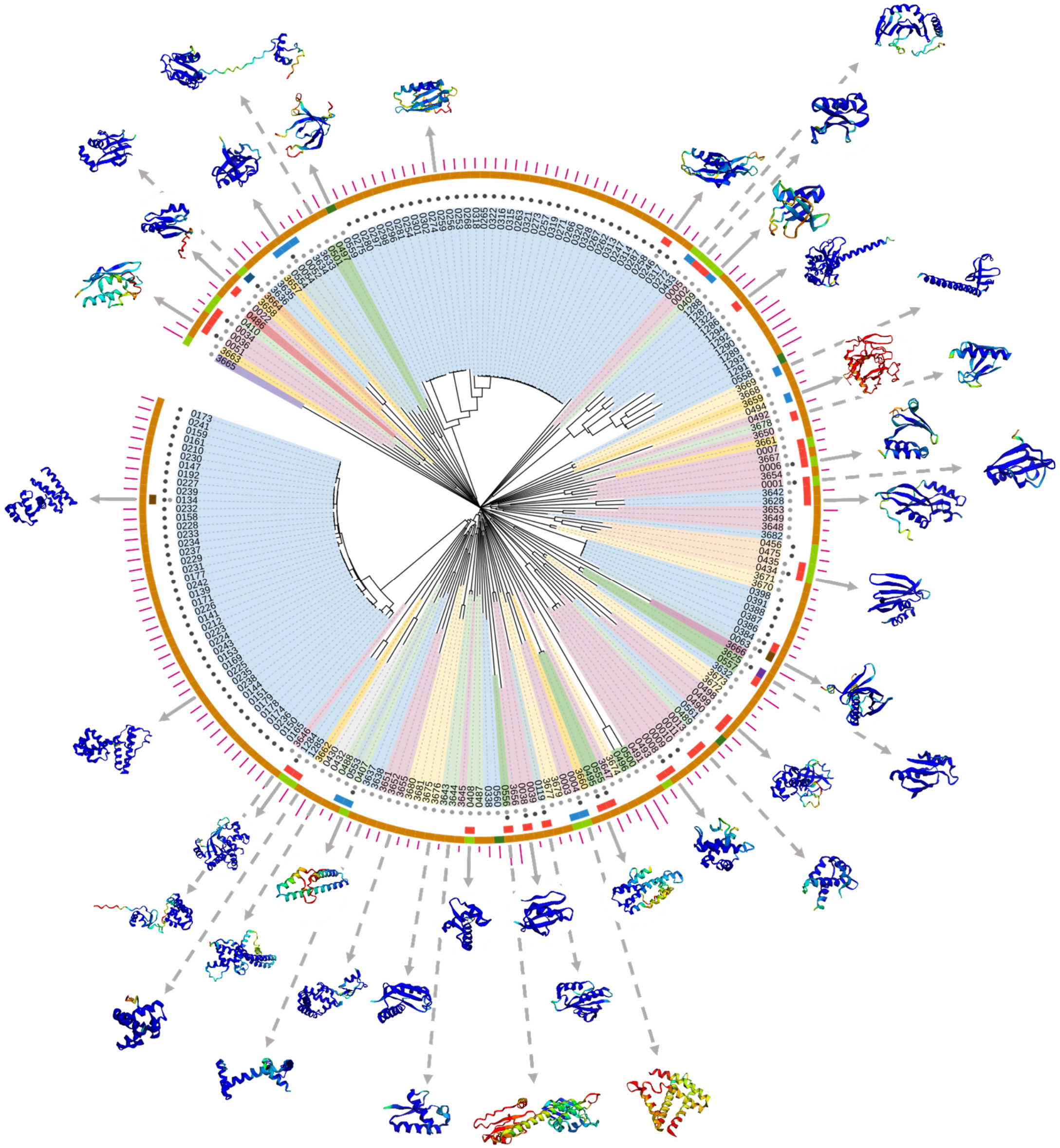

Figure S3. Multiple sequence alignment of anti-CRISPR proteins (Set A + Set B; *n* = 207) using MAFFT, visualized with Jalview. The residue positions are coded with the Taylor protein coloring scheme (Taylor, 1997) using the conservation visibility of 15%.

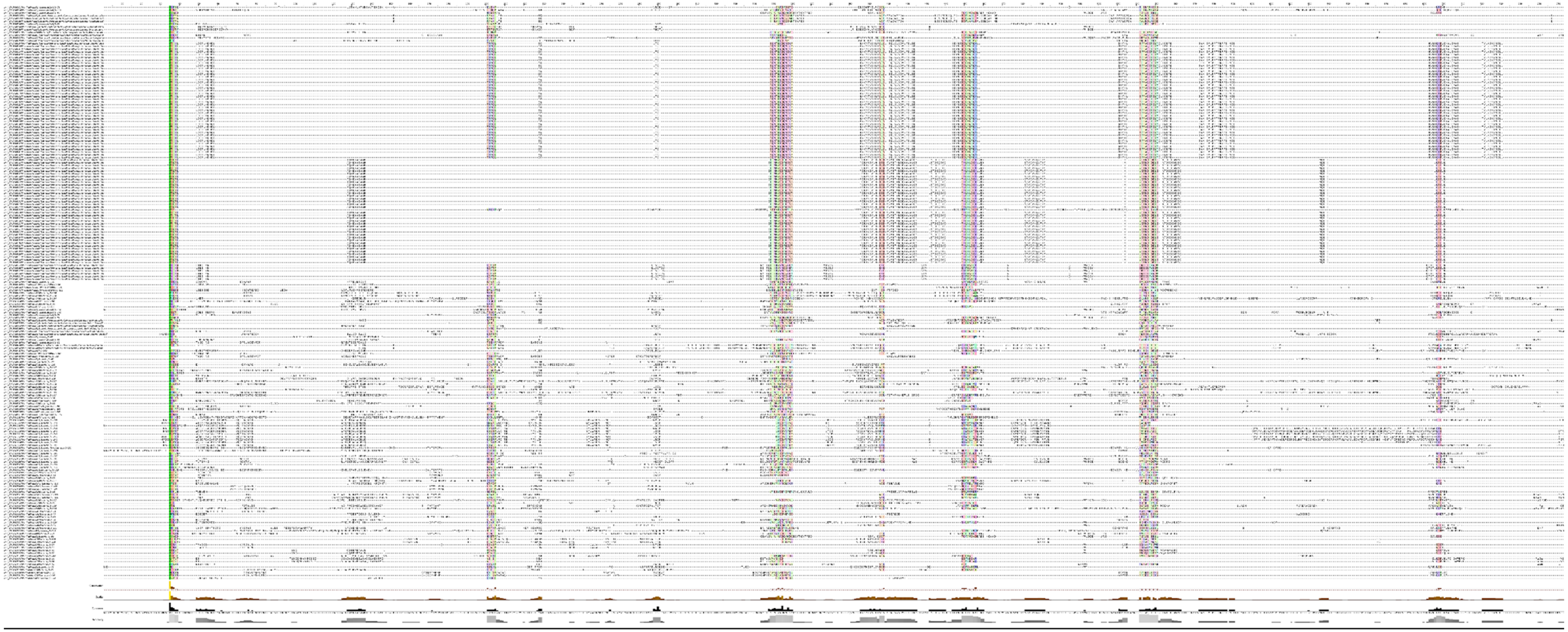

Figure S4. The structural tree of anti-CRISPR proteins reconstructed using structure-based methods (Set A + Set B + Set C; *n* = 443). The AlphaFold-predicted protein structures illustrate representative structural forms for each clade.

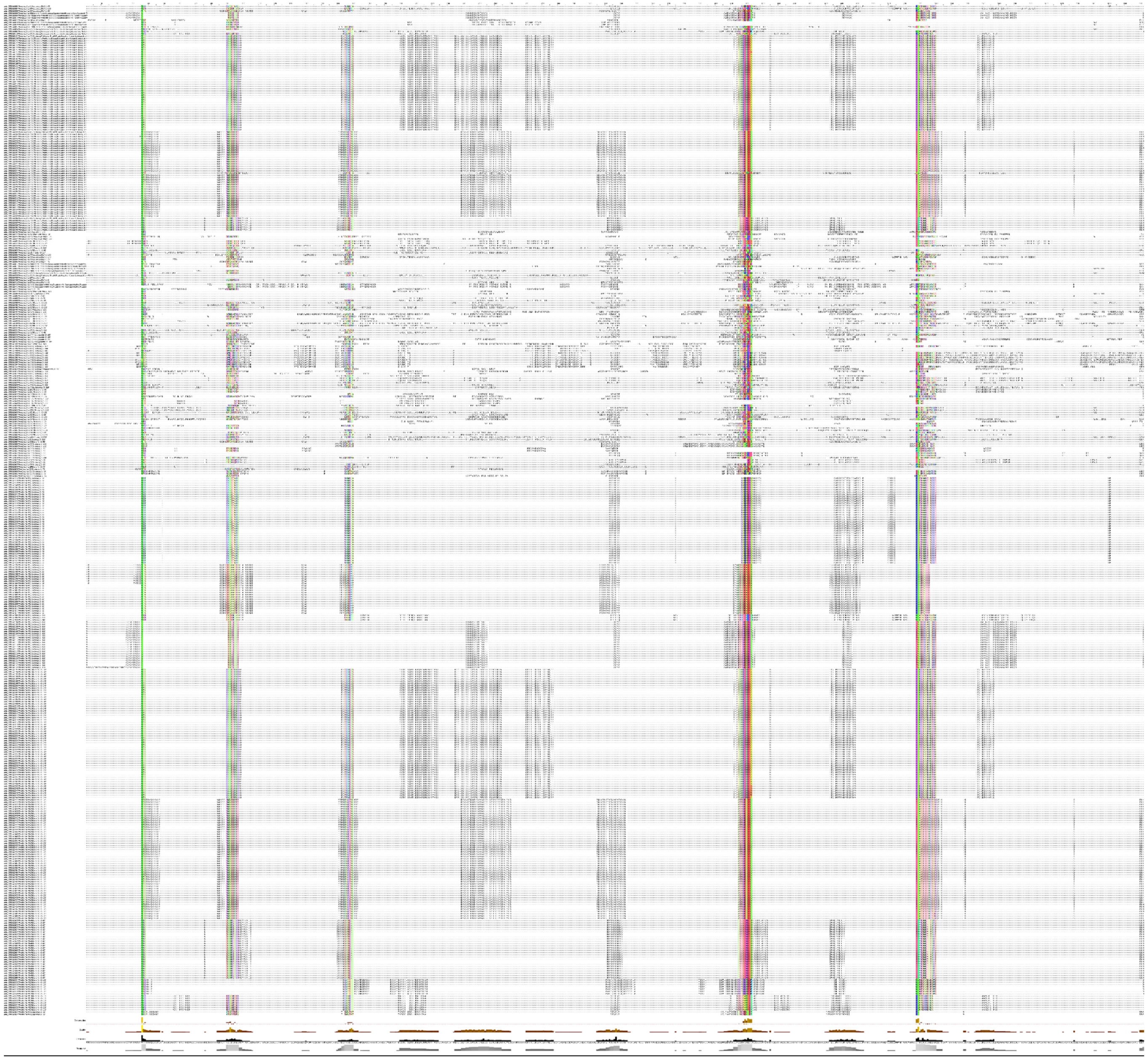

Figure S5. A scatter plot of percentage identity in structure against percentage identity in sequence of the closest homologue to each protein. Some Acr proteins whose % identity in structure is higher than their % identity in sequence with respect to other points of similar % identity in sequence are highlighted in red; those whose % identity in sequence is higher than their % identity in structure with respect to other points of similar % identity in structure are highlighted in purple.

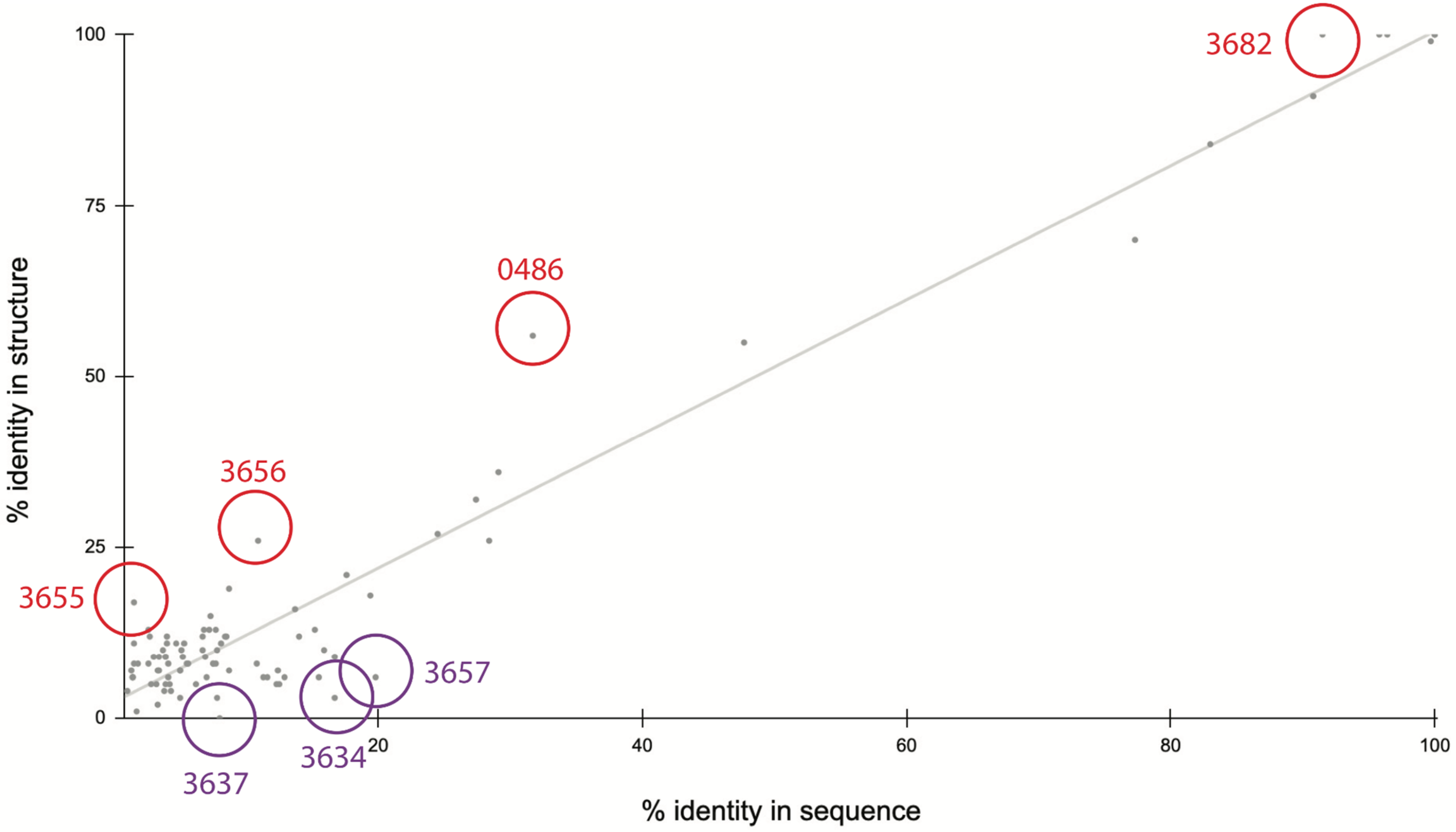

Figure S6. Outlier AlphaFold structures with bad scores (TM-score < 0.8) (**a**), Standalone AlphaFold-predicted structures with pLDDT scores (Set A). (**b**), Superimposition between AlphaFold-predicted structure and true experimental structure with TM-score (Set A). (**c**), Standalone AlphaFold-predicted structures with pLDDT scores (Set C). The closer to red, the lower the confidence of the structure, and the closer to blue, the higher the confidence in the structure of AlphaFold. (**d**), Superimposition between AlphaFold-predicted structure and true experimental structure with TM-score (Set C). For the superimposed image at the bottom, yellow means the ground truth and purple means the predicted structure by AlphaFold.

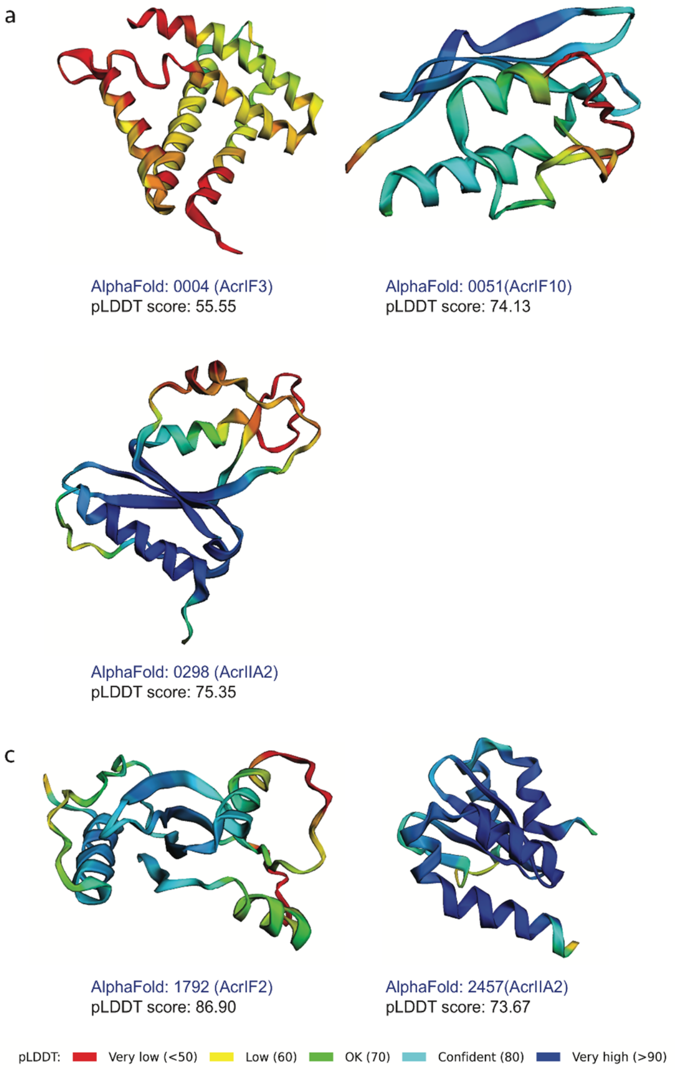

Table S1. Acr protein types in Anti-CRISPRdb.

Type	With Experimental Structure	Without Experimental Structure	Total
**Verified (Database)**	Set A (21)	Set B (98)	119
**Verified (Literature)**	Set A (88)	1,165	1,253
**Putative**	Set C (236)	2,068	2,304
**Total**	345	3,331	3,676

Table S1 is a summary of data published in Anti-CRISPRdb (Dong *et al.*, 2017). The types in the rows are divided into three categories. The first row 'Verified' indicates the protein is validated as CRISPR-Cas-inactivating Acr by the database authors, and the second row 'Literature' shows the protein is likely to be Acr through other published papers. Finally, 'Putative' is the case where the protein was predicted to be Acr but there is no sufficient experimental support to verify the function. The types in the columns indicate whether the protein has a true structure, which is a PDB file from Protein Data Bank (Berman *et al.* 2000) which contains a three-dimensional macromolecular experimental structure. For example, the cell 'Verified' and 'Without experimental structure' shows the number of protein sequences that have been verified as Acr but do not have a true experimental structure (98 cases). In this study, the protein sequences corresponding to 'Verified/Literature' + 'With experimental structure' are denoted as Set A, 'Verified' + 'Without experimental structure' as Set B, and 'Putative' + 'With experimental structure' as Set C.

Table S2. Detailed statistics and scores on AlphaFold prediction performance.

Score Type		TM-score			Relative Z-error			RMSD			plDDT		
Dataset		CASP14	Set A	Set C	CASP14	Set A	Set C	CASP14	Set A	Set C	Set A	Set B	Set C
Count		52	99	207	52	99	207	52	99	207	109	98	236
Mean	Mean	0.882	**0.896**	0.868	0.237	**0.211**	0.259	**1.106**	2.206	2.271	**88.4**	84.6	87.3
	Std	0.120	0.095	**0.092**	0.141	**0.128**	0.129	**0.418**	2.017	1.841	8.4	13.5	**7.8**
Percentiles	Min	0.470	0.409	0.365	0.101	0.102	0.108	0.600	0.582	0.476	55.5	40.4	53.6
	25%	0.868	0.891	0.843	0.165	0.119	0.175	0.745	1.053	1.271	84.4	83.0	83.8
	Median	**0.925**	0.895	0.895	**0.201**	0.217	0.230	**0.980**	1.743	1.560	89.2	**89.4**	89.1
	75%	0.960	0.959	0.922	0.245	0.246	0.296	1.330	1.839	1.887	95.9	92.6	93.9
	Max	0.990	0.984	0.952	0.785	0.883	0.890	2.390	11.824	8.025	96.3	97.2	96.7

Table S3. Closest homologue (highest TM score) to the AlphaFold-predicted structure of Acr proteins with experimentally reconstructed 3-D macromolecular structures (Set A) from the Protein Data Bank archive (downloaded 13 October 2021).

Acr ID	Accession	Family	Type	Organism	Length	Status	Inhibition Stage	PDB Original	Homologue
anti_CRISPR0001	YP_007392342.1	AcrIF1	I-F	Pseudomonas phage JBD30	78	Verified	DNA binding	2LW5;5UZ9;5XLO;5XLP;6ANV;6B46;	6B46_J;5UZ9_J;5UZ9_I;6B46_I;5XLP_M;5XLO_N;2LW5_A;5XLO_M
anti_CRISPR0004	YP_007392739.1	AcrIF3	I-F	Pseudomonas phage JBD5	139	Verified	DNA cleavage	5B7I;5GNF;5GQH;	5B7I_B;5GQH_C;5GQH_B;5GNF_A;5GNF_B;5B7I_C
anti_CRISPR0006	NP_938237.1	AcrIF2	I-F	Pseudomonas phage D3112	90	Verified	DNA binding	5UZ9;5YHR;6B47;	6B47_K
anti_CRISPR0008	WP_043884810.1	AcrIF6	I-E,I-F	Pseudomonas aeruginosa	100	Verified	DNA binding	6VQX	6VQX_A
anti_CRISPR0009	WP_034001826.1	AcrIF6	I-F	Pseudomonas aeruginosa	100	literature		6VQX	6VQX_A
anti_CRISPR0010	WP_031691692.1	AcrIF6	I-F	Pseudomonas aeruginosa	100	literature		6VQX	6VQX_A
anti_CRISPR0034	AFC22483.1	AcrIF8	I-F	Pectobacterium phage ZF40	92	Verified	DNA binding	6VQW	0
anti_CRISPR0038	WP_031500045.1	AcrIF9	I-F	Vibrio parahaemolyticus	68	Verified	DNA binding	6VQV	6VQV_A;6VQV_B
anti_CRISPR0039	EEG86164.1	AcrIF9	I-F	Proteus penneri ATCC 35198	68	literature		6WHI;6W1X	6W1X_J;6WHI_J;6WHI_I;6W1X_I
anti_CRISPR0051	KEK29119.1	AcrIF10	I-F	Shewanella xiamenensis	97	Verified	DNA binding	6B48;6ANW	6ANW_B;6ANW_C;6ANW_A
anti_CRISPR0054	WP_049360089.1	AcrIIC1	II-C	Neisseria meningitidis	85	Verified	DNA cleavage	5VGB	0
anti_CRISPR0063	WP_042743678.1	AcrIIC2	II-C	Neisseria meningitidis	123	Verified	Guide loading	6J9K;6J9L;6J9M;6N05;	0
anti_CRISPR0119	WP_042743676.1	AcrIIC3	II-C	Neisseria meningitidis	116	Verified	DNA binding	6JHV;6JHW;6J9N	0
anti_CRISPR0134	AEO04364.1	AcrIIA1	II-A	Listeria monocytogenes J0161	149	Verified	Guide loading	5Y6A;5Y69	5Y6A_B;5Y6A_A;5Y69_B;5Y69_A
anti_CRISPR0139	AGR27297.1	AcrIIA1	II-A	Listeria monocytogenes	149	literature		5Y69;5Y6A	5Y6A_A;5Y6A_B;5Y69_B;5Y69_A
anti_CRISPR0141	EEW20426.1	AcrIIA1	II-A	Listeria monocytogenes FSL R2-503	149	literature		5Y69;5Y6A	5Y6A_A;5Y6A_B;5Y69_B;5Y69_A
anti_CRISPR0144	EZH69029.1	AcrIIA1	II-A	Listeria monocytogenes N53-1	149	literature		5Y6A	5Y6A_B;5Y6A_A
anti_CRISPR0147	KHK04755.1	AcrIIA1	II-A	Listeria monocytogenes SHL002	149	literature		5Y69;5Y6A	5Y6A_B;5Y6A_A;5Y69_B;5Y69_A
anti_CRISPR0150	KID25720.1	AcrIIA1	II-A	Listeria monocytogenes	149	literature		5Y6A	5Y6A_B;5Y6A_A
anti_CRISPR0151	KKB87492.1	AcrIIA1	II-A	Listeria monocytogenes	149	literature		5Y6A	5Y6A_B;5Y6A_A
anti_CRISPR0153	KTA28092.1	AcrIIA1	II-A	Listeria monocytogenes	149	literature		5Y6A	5Y6A_A;5Y6A_B
anti_CRISPR0158	EAL06505.1	AcrIIA1	II-A	Listeria monocytogenes serotype 1/2a str. F6854	149	literature		5Y69;5Y6A	5Y6A_B;5Y6A_A;5Y69_B;5Y69_A
anti_CRISPR0159	EEW22374.1	AcrIIA1	II-A	Listeria monocytogenes F6900	149	literature		5Y69;5Y6A	5Y6A_A;5Y6A_B;5Y69_B;5Y69_A

anti_CRISPR0161	EFG00298.1	AcrIIA1	II-A	Listeria monocytogenes J2818	149	literature		5Y69;5Y6A	5Y6A_A;5Y6A_B;5Y69_B;5Y69_A
anti_CRISPR0165	KTA33667.1	AcrIIA1	II-A	Listeria monocytogenes	149	literature		5Y69;5Y6A	5Y6A_B;5Y6A_A;5Y69_B;5Y69_A
anti_CRISPR0169	KTA68177.1	AcrIIA1	II-A	Listeria monocytogenes	149	literature		5Y69;5Y6A	5Y6A_A;5Y6A_B;5Y69_B;5Y69_A
anti_CRISPR0171	ALU78083.1	AcrIIA1	II-A	Listeria monocytogenes	149	literature		5Y69;5Y6A	5Y6A_A;5Y6A_B;5Y69_B;5Y69_A
anti_CRISPR0173	KHK19909.1	AcrIIA1	II-A	Listeria monocytogenes SHL008	149	literature		5Y69;5Y6A	5Y6A_A;5Y6A_B;5Y69_B;5Y69_A
anti_CRISPR0174	KHK17523.1	AcrIIA1	II-A	Listeria monocytogenes SHL007	149	literature		5Y69;5Y6A	5Y6A_B;5Y6A_A;5Y69_B;5Y69_A
anti_CRISPR0177	KID20145.1	AcrIIA1	II-A	Listeria monocytogenes	149	literature		5Y6A	5Y6A_B;5Y6A_A
anti_CRISPR0178	KID21568.1	AcrIIA1	II-A	Listeria monocytogenes	149	literature		5Y6A	5Y6A_B;5Y6A_A
anti_CRISPR0179	KID27662.1	AcrIIA1	II-A	Listeria monocytogenes	149	literature		5Y6A	5Y6A_B;5Y6A_A
anti_CRISPR0192	KEU69221.1	AcrIIA1	II-A	Listeria monocytogenes	149	literature		5Y69;5Y6A	5Y6A_B;5Y6A_A;5Y69_B;5Y69_A
anti_CRISPR0210	KTA45326.1	AcrIIA1	II-A	Listeria monocytogenes	149	literature		5Y69;5Y6A	5Y6A_A;5Y6A_B;5Y69_B;5Y69_A
anti_CRISPR0212	KTA50988.1	AcrIIA1	II-A	Listeria monocytogenes	149	literature		5Y69;5Y6A	5Y6A_A;5Y6A_B;5Y69_B;5Y69_A
anti_CRISPR0223	KES96881.1	AcrIIA1	II-A	Listeria monocytogenes	149	literature		5Y69;5Y6A	5Y6A_A;5Y6A_B;5Y69_B;5Y69_A
anti_CRISPR0224	KET73262.1	AcrIIA1	II-A	Listeria monocytogenes	149	literature		5Y69;5Y6A	5Y6A_A;5Y6A_B;5Y69_B;5Y69_A
anti_CRISPR0225	KET94692.1	AcrIIA1	II-A	Listeria monocytogenes	149	literature		5Y69;5Y6A	5Y6A_A;5Y6A_B;5Y69_B;5Y69_A
anti_CRISPR0226	KEV69929.1	AcrIIA1	II-A	Listeria monocytogenes	149	literature		5Y69;5Y6A	5Y6A_A;5Y6A_B;5Y69_B;5Y69_A
anti_CRISPR0227	KEV93281.1	AcrIIA1	II-A	Listeria monocytogenes	149	literature		5Y69;5Y6A	5Y6A_B;5Y6A_A;5Y69_B;5Y69_A
anti_CRISPR0228	KEW08182.1	AcrIIA1	II-A	Listeria monocytogenes	149	literature		5Y69;5Y6A	5Y6A_B;5Y6A_A;5Y69_B;5Y69_A
anti_CRISPR0229	KEW09555.1	AcrIIA1	II-A	Listeria monocytogenes	149	literature		5Y69;5Y6A	5Y6A_B;5Y6A_A;5Y69_B;5Y69_A
anti_CRISPR0230	KEW17020.1	AcrIIA1	II-A	Listeria monocytogenes	149	literature		5Y69;5Y6A	5Y6A_A;5Y6A_B;5Y69_B;5Y69_A
anti_CRISPR0231	KEW65181.1	AcrIIA1	II-A	Listeria monocytogenes	149	literature		5Y69;5Y6A	5Y6A_B;5Y6A_A;5Y69_B;5Y69_A
anti_CRISPR0232	KEX05984.1	AcrIIA1	II-A	Listeria monocytogenes	149	literature		5Y69;5Y6A	5Y6A_B;5Y6A_A;5Y69_B;5Y69_A
anti_CRISPR0233	KHK12400.1	AcrIIA1	II-A	Listeria monocytogenes SHL001	149	literature		5Y69;5Y6A	5Y6A_B;5Y6A_A;5Y69_B;5Y69_A
anti_CRISPR0234	KJJ91612.1	AcrIIA1	II-A	Listeria monocytogenes	149	literature		5Y69;5Y6A	5Y6A_B;5Y6A_A;5Y69_B;5Y69_A
anti_CRISPR0235	KJQ94314.1	AcrIIA1	II-A	Listeria monocytogenes	149	literature		5Y69;5Y6A	5Y6A_A;5Y6A_B;5Y69_B;5Y69_A
anti_CRISPR0236	KJQ95812.1	AcrIIA1	II-A	Listeria monocytogenes	149	literature		5Y69;5Y6A	5Y6A_B;5Y6A_A;5Y69_B;5Y69_A
anti_CRISPR0237	KJR51140.1	AcrIIA1	II-A	Listeria monocytogenes	149	literature		5Y69;5Y6A	5Y6A_B;5Y6A_A;5Y69_B;5Y69_A
anti_CRISPR0238	KJR60209.1	AcrIIA1	II-A	Listeria monocytogenes	149	literature		5Y69;5Y6A	5Y6A_A;5Y6A_B;5Y69_B;5Y69_A

anti_CRISPR0239	KKD43688.1	AcrIIA1	II-A	Listeria monocytogenes	149	literature		5Y69;5Y6A	5Y6A_B;5Y6A_A;5Y69_B;5Y69_A
anti_CRISPR0241	KTA63900.1	AcrIIA1	II-A	Listeria monocytogenes	149	literature		5Y69;5Y6A	5Y6A_A;5Y6A_B;5Y69_B;5Y69_A
anti_CRISPR0242	KXF66381.1	AcrIIA1	II-A	Listeria monocytogenes	149	literature		5Y6A	5Y6A_B;5Y6A_A
anti_CRISPR0243	AGR07061.1	AcrIIA1	II-A	Listeria monocytogenes	149	literature		5Y69;5Y6A	5Y6A_A;5Y6A_B;5Y69_B;5Y69_A
anti_CRISPR0246	AEO04363.1	AcrIIA2	II-A	Listeria monocytogenes J0161	123	Verified	DNA binding	6MCB;6IFO	6IFO_E;6IFO_F;6MCB_C
anti_CRISPR0247	AKI52062.1	AcrIIA2	II-A	Listeria monocytogenes	123	literature		6IFO;6MCB	6IFO_E;6IFO_F;6MCB_C
anti_CRISPR0253	EZH71062.1	AcrIIA2	II-A	Listeria monocytogenes N53-1	124	literature		6IFO;6MCB	0
anti_CRISPR0254	KEU52814.1	AcrIIA2	II-A	Listeria monocytogenes	123	literature		6IFO;6MCB	6IFO_E;6IFO_F;6MCB_C
anti_CRISPR0256	KID23650.1	AcrIIA2	II-A	Listeria monocytogenes	123	literature		6IFO;6MCB	6IFO_E;6IFO_F;6MCB_C
anti_CRISPR0257	KID25721.1	AcrIIA2	II-A	Listeria monocytogenes	123	literature		6IFO;6MCB	6IFO_E;6IFO_F;6MCB_C
anti_CRISPR0258	KKB87491.1	AcrIIA2	II-A	Listeria monocytogenes	123	literature		6IFO;6MCB	6IFO_E;6IFO_F;6MCB_C
anti_CRISPR0259	KKB89544.1	AcrIIA2	II-A	Listeria monocytogenes	123	literature		6IFO;6MCB	6IFO_E;6IFO_F;6MCB_C
anti_CRISPR0262	KXS58607.1	AcrIIA2	II-A	Listeria monocytogenes	123	literature		6IFO;6MCB	6IFO_E;6IFO_F;6MCB_C
anti_CRISPR0263	EAL06504.1	AcrIIA2	II-A	Listeria monocytogenes serotype 1/2a str. F6854	123	literature		6IFO;6MCB	6IFO_E;6IFO_F;6MCB_C
anti_CRISPR0265	EFG00297.1	AcrIIA2	II-A	Listeria monocytogenes J2818	123	literature		6IFO;6MCB	6IFO_E;6IFO_F;6MCB_C
anti_CRISPR0266	KTA33666.1	AcrIIA2	II-A	Listeria monocytogenes	123	literature		6IFO;6MCB	6IFO_E;6IFO_F;6MCB_C
anti_CRISPR0267	KXS56902.1	AcrIIA2	II-A	Listeria monocytogenes	123	literature		6IFO;6MCB	6IFO_E;6MCB_C
anti_CRISPR0268	KXW85500.1	AcrIIA2	II-A	Listeria monocytogenes	122	literature		6IFO;6MCB	6IFO_E;6IFO_F;6MCB_C
anti_CRISPR0271	KID20146.1	AcrIIA2	II-A	Listeria monocytogenes	123	literature		6IFO;6MCB	6IFO_E;6IFO_F;6MCB_C
anti_CRISPR0272	KID21567.1	AcrIIA2	II-A	Listeria monocytogenes	123	literature		6IFO;6MCB	6IFO_E;6IFO_F;6MCB_C
anti_CRISPR0273	KID27661.1	AcrIIA2	II-A	Listeria monocytogenes	123	literature		6IFO;6MCB	6IFO_E;6IFO_F;6MCB_C
anti_CRISPR0274	KXX34834.1	AcrIIA2	II-A	Listeria monocytogenes	123	literature		6IFO;6MCB	6IFO_E;6IFO_F;6MCB_C
anti_CRISPR0275	KXX34219.1	AcrIIA2	II-A	Listeria monocytogenes	130	literature		6MCC	6MCC_C
anti_CRISPR0280	KES29690.1	AcrIIA2	II-A	Listeria monocytogenes	123	literature		6IFO;6MCB	6IFO_E;6IFO_F;6MCB_C
anti_CRISPR0281	KES36190.1	AcrIIA2	II-A	Listeria monocytogenes	123	literature		6IFO;6MCB	6IFO_E;6IFO_F;6MCB_C
anti_CRISPR0287	KEU69222.1	AcrIIA2	II-A	Listeria monocytogenes	123	literature		6IFO;6MCB	6IFO_E;6IFO_F;6MCB_C
anti_CRISPR0296	KEX13879.1	AcrIIA2	II-A	Listeria monocytogenes	123	literature		6IFO;6MCB	6IFO_E;6IFO_F;6MCB_C
anti_CRISPR0297	KEX45732.1	AcrIIA2	II-A	Listeria monocytogenes	123	literature		6IFO;6MCB	6IFO_E;6IFO_F;6MCB_C

anti_CRISPR0298	KEX49272.1	AcrIIA2	II-A	Listeria monocytogenes	123	literature		6IFO;6MCB	6IFO_E;6IFO_F;6MCB_C
anti_CRISPR0301	KLI12475.1	AcrIIA2	II-A	Listeria monocytogenes	123	literature		6IFO;6MCB	6IFO_E;6IFO_F;6MCB_C
anti_CRISPR0302	KNX95906.1	AcrIIA2	II-A	Listeria monocytogenes	123	literature		6IFO;6MCB	6IFO_E;6IFO_F;6MCB_C
anti_CRISPR0313	KES96882.1	AcrIIA2	II-A	Listeria monocytogenes	123	literature		6IFO;6MCB	6IFO_E;6IFO_F;6MCB_C
anti_CRISPR0314	KET73263.1	AcrIIA2	II-A	Listeria monocytogenes	123	literature		6IFO;6MCB	6IFO_E;6IFO_F;6MCB_C
anti_CRISPR0315	KET94691.1	AcrIIA2	II-A	Listeria monocytogenes	123	literature		6IFO;6MCB	6IFO_E;6IFO_F;6MCB_C
anti_CRISPR0316	KEV69928.1	AcrIIA2	II-A	Listeria monocytogenes	123	literature		6IFO;6MCB	6IFO_E;6IFO_F;6MCB_C
anti_CRISPR0317	KEV93282.1	AcrIIA2	II-A	Listeria monocytogenes	123	literature		6IFO;6MCB	6IFO_E;6IFO_F;6MCB_C
anti_CRISPR0318	KEW08181.1	AcrIIA2	II-A	Listeria monocytogenes	123	literature		6IFO;6MCB	6IFO_E;6IFO_F;6MCB_C
anti_CRISPR0319	KEW09554.1	AcrIIA2	II-A	Listeria monocytogenes	123	literature		6IFO;6MCB	6IFO_E;6IFO_F;6MCB_C
anti_CRISPR0320	KEW17021.1	AcrIIA2	II-A	Listeria monocytogenes	123	literature		6IFO;6MCB	6IFO_E;6IFO_F;6MCB_C
anti_CRISPR0321	KEW65182.1	AcrIIA2	II-A	Listeria monocytogenes	123	literature		6IFO;6MCB	6IFO_E;6IFO_F;6MCB_C
anti_CRISPR0322	KEX05985.1	AcrIIA2	II-A	Listeria monocytogenes	123	literature		6IFO;6MCB	6IFO_E;6IFO_F;6MCB_C
anti_CRISPR0328	KXF66382.1	AcrIIA2	II-A	Listeria monocytogenes	123	literature		6IFO;6MCB	6IFO_E;6IFO_F;6MCB_C
anti_CRISPR0384	AEO04689.1	AcrIIA4	II-A	Listeria monocytogenes J0161	87	Verified	DNA binding	5XN4;5VZL;5VW1	5VZL_C;5VW1_B;5XN4_X
anti_CRISPR0386	EAL05809.1	AcrIIA4	II-A	Listeria monocytogenes serotype 1/2a str. F6854	87	literature		5VW1;5VZL;5XBL;5XN4	5VZL_C;5VW1_B;5XN4_X;5XBL_D
anti_CRISPR0387	EEW23439.1	AcrIIA4	II-A	Listeria monocytogenes F6900	87	literature		5VW1;5VZL;5XBL;5XN4	5VZL_C;5VW1_B;5XN4_X;5XBL_D
anti_CRISPR0388	EFG00182.1	AcrIIA4	II-A	Listeria monocytogenes J2818	87	literature		5VW1;5VZL;5XBL;5XN4	5VZL_C;5VW1_B;5XN4_X;5XBL_D
anti_CRISPR0391	KTA31189.1	AcrIIA4	II-A	Listeria monocytogenes	87	literature		5VW1;5VZL;5XBL;5XN4	5VZL_C;5VW1_B;5XN4_X;5XBL_D
anti_CRISPR0398	AMD24318.1	AcrIIA4	II-A	Listeria monocytogenes	87	literature		5VW1;5VZL;5XBL;5XN4	5VZL_C;5VW1_B;5XN4_X;5XBL_D
anti_CRISPR0407	YP_007392738.1	AcrIE1	I-E	Pseudomonas phage JBD5	100	Verified	DNA cleavage	6ARZ;6AS3;6AS4;	6ARZ_B;6ARZ_A;6ARZ_C
anti_CRISPR0434	YP_009272954.1	AcrID1	I-D	Sulfolobus islandicus rudivirus 3	96	Verified	DNA binding	6EXP;	6EXP_D;6EXP_E;6EXP_B;6EXP_C;6EXP_A;6EXP_F
anti_CRISPR0456	YP_003728.1	AcrID1	I-D	Acidianus filamentous virus 1	99	literature		3DF6;3DJW;	3DJW_B;3DJW_A
anti_CRISPR0475	NP_445679.1	AcrID1	I-D	Sulfolobus islandicus filamentous virus	112	literature		2H36;	0
anti_CRISPR0495	AKG19227.1	AcrVA1	V-A	Moraxella bovoculi	170	Verified	DNA binding	6NMD	0
anti_CRISPR0555	WP_046701302.1	AcrVA1	V-A	Moraxella bovoculi	170	Verified	DNA binding	6NMD	0

anti_CRISPR0556	WP_046699156.1	AcrVA4	V-A	Moraxella bovoculi	234	Verified	DNA binding	6KLB; 6KL9; 6P7N; 6P7M; 6NM9; 6OMV; 6NMA	6KL9_B;6KLB_B;6KL9_C;6NM9_A;6NMA_C;6NM9_C;6NMA_A;6OMV_A;6KLB_C;6OMV_C
anti_CRISPR1284	AVO22749.1	AcrIIA6	II-A	Streptococcus phage D1811	183	Verified	DNA binding	6RJ9;6RJA;6RJG	6RJG_A;6RJ9_B;6RJ9_A;6RJG_B;6RJA_A;6RJA_B
anti_CRISPR3625	AKG12174.1	AcrVA5	V-A	Moraxella bovoculi	92	Verified	DNA binding (acetylation)	6IUF	6IUF_B
anti_CRISPR3664	NP_666617.1	AcrIII1	III-1	Sulfolobus islandicus rod-shaped virus 1	114	Verified	Degrad cA4	2X4I;6SCF	6SCF_A;2X4I_C;2X4I_A;2X4I_D;6SCF_H

Table S4. Closest homologue (highest DALI Z-score) to the AlphaFold-predicted structure of Acr proteins without experimentally reconstructed 3-D macromolecular structures (Set B) from the Protein Data Bank archive (downloaded 13 October 2021). Acr proteins with the N/A rows indicate the absence of homologues retrieved from the PDB archive using DaliLite.v5 with the Z-score threshold above 2.

Acr ID	Accession	Family	Type	Organism	Length	Homologue	DALI Z-score	PDB annotation	Classification	Organism	%ID structure	%ID seq
anti_CRISPR0002	YP_007392799.1	AcrIF4	I-F	Pseudomonas phage JBD24	100	7jzw-J	11.4	AcrIF4	AcrIF	Pseudomonas aeruginosa, Pseudomonas phage sp.	100	100
anti_CRISPR0003	YP_007392440.1	AcrIF3	I-F	Pseudomonas phage JBD88a	140	5b7i-C	8	AcrIF3	AcrIF	Pseudomonas aeruginosa UCBPP-PA14, Pseudomonas phage JBD5	70	77.3
anti_CRISPR0005	YP_007392740.1	AcrIF5	I-F	Pseudomonas phage JBD5	79	3bzc-A	6.2	Tex (toxin expression)	Toxin	Pseudomonas aeruginosa	13	2.6
anti_CRISPR0007	YP_002332454.1	AcrIF2	I-F	Pseudomonas phage MP29	91	5o9j-A	3.3	Transcription factor IIB Mja mini-intein	Hydrolase	Methanocaldococcus jannaschii DSM 2661	2	3.3
anti_CRISPR0011	WP_019933870.1	AcrIF6	I-F	Oceanimonas smirnovii	95	6vqx-A	11.9	AcrIF6	AcrIF	Pseudomonas aeruginosa	36	29.1
anti_CRISPR0013	WP_014702809.1	AcrIF6	I-F	Methylophaga frappieri	85	6vqx-A	12.3	AcrIF6	AcrIF	Pseudomonas aeruginosa	26	28.4
anti_CRISPR0022	ACD38920.1	AcrIF7	I-F	Pseudomonas aeruginosa	83	7jzx-J	12.1	AcrIF4	AcrIF	Pseudomonas aeruginosa	100	100
anti_CRISPR0036	ACD38920.1	AcrIF7	I-F	Pseudomonas aeruginosa	83	N/A	N/A	N/A	N/A	N/A	N/A	N/A
anti_CRISPR0052	KEH13790.1	AcrIF8	I-F	Delftia sp. 670	81	6yes-A	3.5	Cas10d Type ID	Cas	Sulfolobus islandicus LAL14/1	1	1.7
anti_CRISPR0338	EXL25968.1	AcrIIA3	II-A	Listeria monocytogenes Lm_1880	125	6n8z-E	5.7	Heat shock protein 104	Chaperone	Saccharomyces cerevisiae S288C	12	2.7
anti_CRISPR0408	YP_950454.1	AcrIE3	I-E	Pseudomonas phage DMS3	68	5aj3-b	4	Mitoribosomal Protein US2M, MRPS2	Ribosome	Sus scrofa	10	5.1
anti_CRISPR0409	YP_007392439.1	AcrIE2	I-E	Pseudomonas phage JBD88a	84	4fbr-A	4.5	Myxococcus Xanthus hemagglutinin (MBHA)	Carbohydrate binding protein	Myxococcus xanthus	8	4.1
anti_CRISPR0410	NP_938238.1	AcrIE4	I-E	Pseudomonas phage D3112	52	1zp9-C	2.7	Rio1 Serine Kinase bound to ATP and Mn2+ ions.	Kinase	Archaeoglobus fulgidus DSM 4304	3	5

anti_CRISPR0430	EKB54194.1	AcrVIB	VI-B	Bergeyella zoohelcum ATCC 43767	219	6qv0-C	6.3	ABC transporter, ATP-binding protein	Membrane protein	Thermotoga maritima MSB8, synthetic construct	13	7.7
anti_CRISPR0432	WP_034985946.1	AcrVIB	VI-B	Bergeyella zoohelcum	201	6qv0-C	8.4	ABC transporter, ATP-binding protein	Membrane protein	Thermotoga maritima MSB8, synthetic construct	13	7.2
anti_CRISPR0433	ASD50988.1	AcrIIA5	II-A	Streptococcus phage D4276	140	1cw0-A	3.7	DNA Mismatch Endonuclease	Nuclease	Escherichia coli	7	8.7
anti_CRISPR0435	NP_666537.1	AcrID1	I-D	Sulfolobus islandicus rod-shaped virus 2	103	6exp-D	17.3	AcrID5	AcrID	Sulfolobus islandicus rudivirus 3	55	47.7
anti_CRISPR0486	WP_064584002.1	AcrIE4-F7	I-E,I-F	Pseudomonas citronellolis	119	7jzx-J	13.5	AcrIF4	AcrIF	Pseudomonas aeruginosa	56	31.7
anti_CRISPR0487	WP_074973300.1	AcrIE5	I-E	Pseudomonas otitidis	65	6h4c-H	4.3	Dutpase	dUTP diphosphatase	Staphylococcus virus 11	12	4
anti_CRISPR0488	WP_087937214.1	AcrIE6	I-E	Pseudomonas aeruginosa	79	3onl-C	8.8	t-SNARE VTI1	Protein transport	Saccharomyces cerevisiae	7	12.4
anti_CRISPR0489	WP_087937215.1	AcrIE7	I-E	Pseudomonas aeruginosa	106	5dn6-H	5.7	ATP synthase subunit delta	Hydrolase	Paracoccus denitrificans	6	12.9
anti_CRISPR0490	WP_038819808.1	AcrIF11	I-F	Pseudomonas aeruginosa	132	6kyf-A	24.4	AcrIF11	AcrIF	Pseudomonas aeruginosa	100	96.4
anti_CRISPR0491	ABR13388.1	AcrIF12	I-F	Pseudomonas aeruginosa	124	3t49-B	5	Fibrinogen-binding protein	Immune system	Staphylococcus aureus subsp. aureus Mu50	6	11.3
anti_CRISPR0492	EGE18854.1	AcrIF13	I-F	Moraxella catarrhalis BC8	115	3ibj-B	4.3	cGMP-dependent 3',5'-cyclic phosphodiesterase	Hydrolase	Homo sapiens	7	3.4
anti_CRISPR0493	AKI27193.1	AcrIF14	I-F	Moraxella phage Mcat5	124	7jzz-K	9.3	AcrIF14	AcrIF	Pseudomonas aeruginosa, unidentified	100	100
anti_CRISPR0494	AKG19229.1	AcrIC1	I-C	Moraxella bovoculi	190	4ggt-B	3.5	Bradavidin 2	Biotin binding protein	Bradyrhizobium diazoefficiens USDA 110	8	5.6
anti_CRISPR0496	AKG19228.1	AcrVA2	V-A	Moraxella bovoculi	322	7ci2-A	41.4	AcrVA2	AcrVA	Moraxella bovoculi	99	99.7
anti_CRISPR0497	AKG19230.1	AcrVA3	V-A	Moraxella bovoculi	168	1u3e-M	4.8	HNH homing endonuclease	Nuclease	Bacillus virus SPO1	12	14
anti_CRISPR0498	WP_033936089.1	AcrIF11	I-F	Pseudomonas aeruginosa	152	6kyf-A	12.2	AcrIF11	AcrIF	Pseudomonas aeruginosa	27	24.5
anti_CRISPR0499	EGE18857.1	AcrIF11	I-F	Moraxella catarrhalis BC8	156	6kyf-A	15.3	AcrIF11	AcrIF	Pseudomonas aeruginosa	32	27.4
anti_CRISPR0500	AKG12143.1	AcrVA2	V-A	Moraxella bovoculi	319	7ci2-A	41.5	AcrVA2	AcrVA	Moraxella bovoculi	84	83
anti_CRISPR0501	OOR90252.1	AcrVA3	V-A,I-C	Moraxella caviae	167	1u3e-M	6.1	HNH homing endonuclease	Nuclease	Bacillus virus SPO1	12	8.4

anti_CRISPR0553	WP_049372635.1	AcrIIC4	II-C	Haemophilus parainfluenzae	88	4brr-C	7.3	Diacylglycerol kinase	Kinase	Escherichia coli K-12	6	11.6
anti_CRISPR0554	WP_002642161.1	AcrIIC5	II-C	Simonsiella muelleri	130	3rpx-B	3	Complement component 1 Q subcomponent-binding protein	Protein binding	Homo sapiens	9	16.7
anti_CRISPR0557	WP_046699157.1	AcrVA5	V-A	Moraxella bovoculi	92	6iuf-B	19.7	AcrVA5	AcrVA	Moraxella bovoculi	100	95.8
anti_CRISPR0558	VDB32354.1	AcrIIA7	II-A	metagenome	103	6j6h-g	3.8	Small nuclear ribonucleoprotein Sm D2	Splicing	Saccharomyces cerevisiae S288C	6	15.5
anti_CRISPR0559	VDB32352.1	AcrIIA8	II-A	metagenome	105	6te9-E	9	Stopper protein Rcc01689	Viral particle	Rhodobacter capsulatus	6	1.4
anti_CRISPR0560	VDB32351.1	AcrIIA9	II-A	metagenome	141	4cht-B	5.2	RecQ-mediated genome instability protein 1	Nucleotide binding	Homo sapiens	3	7.8
anti_CRISPR0561	VDB32353.1	AcrIIA10	II-A	metagenome	109	4icg-D	4.6	Hemolysin expression modulating protein (environmental regulation of virulence factors)	DNA binding, Chaperone	Salmonella enterica subsp. enterica serovar Typhimurium str. LT2	15	7.3
anti_CRISPR1285	AVO22721.1	AcrIIA6	II-A	Streptococcus phage D1024	183	6eyx-A	26	AcrIIA6	AcrIIA	Streptococcus virus DT1	91	90.8
anti_CRISPR1286	WP_064786071.1	AcrIIA11	II-A	Clostridiales	181	4h63-Q	6	Mediator of RNA polymerase II transcription subunit 17	Polymerase	Schizosaccharomyces pombe 972h-	12	8.5
anti_CRISPR1287	OHE28210.1	AcrIIA11	II-A	Tenericutes bacterium GWC2_34_14 (subsurface metagenome)	144	5oqm-d	5.2	DNA-directed RNA polymerase II subunit RPB4	Polymerase	Saccharomyces cerevisiae S288C, synthetic construct, Saccharomyces cerevisiae	5	3.9
anti_CRISPR1288	OHE43765.1	AcrIIA11	II-A	Tenericutes bacterium RIFOXYA2_FULL_36_32 (subsurface metagenome)	145	7pdz-F	5.2	F-actin-capping protein subunit alpha-1	Structural protein	Mus musculus, Bos taurus, synthetic construct	8	1.5
anti_CRISPR1289	WP_006572312.1	AcrIIA11	II-A	Pseudoflavonifractor capillosus	244	5oqm-d	6.1	DNA-directed RNA polymerase II subunit RPB4	Polymerase	Saccharomyces cerevisiae S288C, synthetic construct, Saccharomyces cerevisiae	7	1.3

anti_CRISPR1290	WP_009258904.1	AcrIIA11	II-A	Flavonifractor plautii	245	5oqm-d	6.2	DNA-directed RNA polymerase II subunit RPB4	Polymerase	Saccharomyces cerevisiae S288C, synthetic construct, Saccharomyces cerevisiae	10	3.7
anti_CRISPR1291	WP_054338718.1	AcrIIA11	II-A	Clostridia bacterium UC5.1-2H11	242	5oqm-d	6.1	DNA-directed RNA polymerase II subunit RPB4	Polymerase	Saccharomyces cerevisiae S288C, synthetic construct, Saccharomyces cerevisiae	6	4.1
anti_CRISPR1292	WP_016321673.1	AcrIIA11	II-A	Oscillibacter sp. 1-3	249	3nct-B	6.3	Protein psiB	DNA binding, Chaperone	Escherichia coli	8	10.8
anti_CRISPR1293	WP_023346767.1	AcrIIA11	II-A	Firmicutes bacterium ASF500	249	5oqm-d	6.3	DNA-directed RNA polymerase II subunit RPB4	Polymerase	Saccharomyces cerevisiae S288C, synthetic construct, Saccharomyces cerevisiae	9	3
anti_CRISPR1294	WP_055271317.1	AcrIIA11	II-A	Flavonifractor plautii	165	5oqm-d	5.5	DNA-directed RNA polymerase II subunit RPB4	Polymerase	Saccharomyces cerevisiae S288C, synthetic construct, Saccharomyces cerevisiae	5	4.2
anti_CRISPR1322	WP_118651841.1	AcrIIA11	II-A	Clostridium	182	4h63-Q	5.8	Mediator of RNA polymerase II transcription subunit 17	Polymerase	Schizosaccharomyces pombe 972h-	11	8.1
anti_CRISPR3628	WP_050337628.1	AcrIIA13	II-A	Staphylococcus schleiferi	131	6q0w-C	5.1	DDB1- and CUL4-associated factor 15	Ligase	Homo sapiens	5	12.5
anti_CRISPR3632	WP_025188019.1	AcrIIA16	II-A	Enterococcus faecalis	204	7a0r-M	3.5	50S ribosomal protein L19	Ribosome	Deinococcus radiodurans R1	11	4.7
anti_CRISPR3633	WP_002401839.1	AcrIIA17	II-A	Enterococcus faecalis	109	7n1n-A	4.8	Prx (Paratox)	Toxin	Streptococcus pyogenes MGAS315, Streptococcus mutans UA159	19	8.7
anti_CRISPR3634	WP_074626943.1	AcrIIA17	II-A	Streptococcus gallolyticus	100	5ld9-A	3.5	JAMM1	Hydrolase	Pyrococcus furiosus DSM 3638	3	16.7
anti_CRISPR3635	WP_099390844.1	AcrIIA18	II-A	Streptococcus macedonicus	182	5jm7-A	2.7	Aerobactin synthase IucA	Ligase	Klebsiella pneumoniae subsp. pneumoniae	9	6.9
anti_CRISPR3636	WP_074627086.1	AcrIIA18	II-A	Streptococcus gallolyticus	181	5jm7-A	3.8	Aerobactin synthase IucA	Ligase	Klebsiella pneumoniae subsp. pneumoniae	9	5.2
anti_CRISPR3637	WP_107591702.1	AcrIIA19	II-A	Staphylococcus simulans	124	3vyg-H	4.6	Thiocyanate hydrolase subunit beta	Hydrolase	Thiobacillus thioparus	0	8
anti_CRISPR3638	WP_100006909.1	AcrIIA19	II-A	Staphylococcus pseudintermedius	115	N/A	N/A	N/A	N/A	N/A	N/A	N/A

anti_CRISPR3642	WP_053038109.1	AcrIIA13b	II-A	Staphylococcus haemolyticus	129	6q0w-C	5.1	DDB1- and CUL4-associated factor 15	Ligase	Homo sapiens	5	12.3
anti_CRISPR3643	WP_117085605.1	AcrIE8.2	I-E	Klebsiella pneumoniae	63	6hzg-A	4.6	BP0997, GH138 enzyme targeting pectin rhamnogalacturonan II	Hydrolase	Phocaeicola paurosaccharolyticus	8	1.8
anti_CRISPR3644	WP_038434996.1	AcrIE8.1	I-E	Klebsiella pneumoniae	63	6hzg-A	4.7	BP0997, GH138 enzyme targeting pectin rhamnogalacturonan II	Hydrolase	Phocaeicola paurosaccharolyticus	11	1.5
anti_CRISPR3645	WP_117085604.1	AcrIF15	I-F	Klebsiella pneumoniae	69	6blm-B	5.2	4-oxalocrotonate tautomerase	Hydrolase	Burkholderia lata	5	6.2
anti_CRISPR3646	WP_121296237.1	AcrIF16	I-F	Pectobacterium parmentieri	171	1rio-A	6.2	Repressor protein CI	Regulatory	Thermus aquaticus, Escherichia virus Lambda	9	3.4
anti_CRISPR3647	WP_102117861.1	AcrIF17.1	I-F	Pectobacterium carotovorum (Erwinia carotovora)	116	4akg-A	4	Glutathione S-transferase	Motor protein	Schistosoma japonicum	4	1
anti_CRISPR3648	WP_049300010.1	AcrIF18.1	I-F/I-E	Serratia marcescens	69	6vc7-F	5.4	Periplasmic domain of cardiolipin transporter protein YejM/PbgA	Transport protein	Salmonella enterica subsp. enterica serovar Typhimurium	9	3.9
anti_CRISPR3649	WP_060431798.1	AcrIF18.2	I-F/I-E	Serratia marcescens	69	6vc7-F	5.3	Periplasmic domain of cardiolipin transporter protein YejM/PbgA	Transport protein	Salmonella enterica subsp. enterica serovar Typhimurium	9	3.9
anti_CRISPR3650	WP_119870654.1	AcrIF19	I-F	Pectobacterium	93	6cqi-A	4.3	DNA topoisomerase 1	Topoisomerase	Mycobacterium tuberculosis H37Rv	5	2.8
anti_CRISPR3651	WP_119870655.1	AcrIF20.1	I-F	Pectobacterium	121	7mfl-A	6.6	Alpha-N-acetylglucosaminidase family protein	Acetylglucosaminidase	Clostridium perfringens ATCC 13124	7	3.3
anti_CRISPR3652	WP_121268706.1	AcrIF20.2	I-F	Pectobacterium parmentieri	121	5mm0-A	4.6	Dolichol monophosphate mannose synthase	Membrane protein	Pyrococcus furiosus DSM 3638	6	7

anti_CRISPR3653	WP_102117862.1	AcrIF21	I-F	Pectobacterium carotovorum (Erwinia carotovora)	162	1x6o-A	3.7	Eukaryotic initiation factor 5a	Regulatory	Leishmania braziliensis	10	7.8
anti_CRISPR3654	WP_109463511.1	AcrIF22	I-F/I-E	Pectobacterium parmentieri	88	6b46-J	6	AcrIF	AcrIF	Pseudomonas aeruginosa UCBPP-PA14, Pseudomonas phage JBD30, Pseudomonas aeruginosa	18	19.4
anti_CRISPR3655	WP_052155777.1	AcrIF23	I-F	Pseudomonas aeruginosa	159	4rqi-B	6.4	Telomeric repeat-binding factor 2	Regulatory	Homo sapiens	17	1.5
anti_CRISPR3656	WP_043084540.1	AcrIF24	I-F	Pseudomonas aeruginosa	228	7vjm-A	8.3	Aca1	Acr-related	Pseudomonas phage JBD30, synthetic construct	26	10.9
anti_CRISPR3657	ERK51680.1	AcrVIA1	VI-A	Leptotrichia wadei F0279	74	1ywl-A	3.7	Hypothetical protein	Unknown	Enterococcus faecalis	6	19.8
anti_CRISPR3658	ERK51681.1	AcrVIA2	VI-A	Leptotrichia wadei F0279	77	N/A	N/A	N/A	N/A	N/A	N/A	N/A
anti_CRISPR3659	ERK48335.1	AcrVIA3	VI-A	Leptotrichia wadei F0279	86	7bjd-A	3.8	Serine/threonine-protein kinase Chk1Serine/threonine-protein kinase Chk1Serine/threonine-protein kinase Chk1	Kinase	Homo sapiens	8	5.5
anti_CRISPR3660	ERK48333.1	AcrVIA4	VI-A	Leptotrichia wadei F0279	76	6q0t-X	6	14-3-3 protein zeta	Transferase/Protein binding	Homo sapiens, Spodoptera exigua	11	5.3
anti_CRISPR3661	ERK48092.1	AcrVIA5	VI-A	Leptotrichia wadei F0279	124	5m3l-N	4.4	Extracellular hemoglobin linker L2 subunit	Transport protein	Lumbricus terrestris	4	4.3
anti_CRISPR3662	ETD74580.1	AcrVIA6	VI-A	Rhodobacter capsulat R121	85	4i6u-D	8	Regulatory protein	Regulatory	Enterobacter sp. RFL1396	11	4
anti_CRISPR3663	ACV38861.1	AcrVIA7	VI-A	Leptotrichia buccalis C-1013-b	151	6y8q-D	7	Abortive phage infection protein	Toxin	Streptococcus agalactiae	13	15.2
anti_CRISPR3665	NP_666582.1	AcrIIIB1	III-B	Sulfolobus islandicus rod-shaped virus 2	249	5xgp-A	3.7	Secreted Xwnt8 inhibitor sizzled	Unknown	Xenopus laevis	8	7.7
anti_CRISPR3666	ACV38859.1	AcrIB	I-B	Leptotrichia buccalis C-1013-b	193	7ak8-B	16.8	Acetyltransferase	Toxin	Salmonella enterica subsp. enterica serovar Typhimurium	21	17.6
anti_CRISPR3667	WP_015972868.1	AcrIF2	I-C/I-F	Pseudomonas aeruginosa	90	5yww-A	4.6	Nucleotide binding protein PINc	Hydrolase	Sulfolobus islandicus REY15A	5	3.2

anti_CRISPR3668	WP_058130594.1	AcrIC3	I-C	Pseudomonas aeruginosa	100	4iap-A	5.8	Oxysterol-binding protein homolog 3,Endolysin,Oxysterol-binding protein homolog 3	Lipid binding protein	Saccharomyces cerevisiae S288C, Escherichia virus T4	8	7.5
anti_CRISPR3669	KSR23770.1	AcrIC3	I-C	Pseudomonas aeruginosa	100	4iap-A	5.8	Oxysterol-binding protein homolog 3,Endolysin,Oxysterol-binding protein homolog 3	Lipid binding protein	Saccharomyces cerevisiae S288C, Escherichia virus T4	8	7.5
anti_CRISPR3670	WP_153575361.1	AcrIC4	I-C	Pseudomonas aeruginosa	57	6qss-D	4.1	Malate dehydrogenase	Oxidoreductase	Ignicoccus islandicus DSM 13165	12	6.7
anti_CRISPR3671	CDO85538.1	AcrIC4	I-C	Pseudomonas aeruginosa	57	6qss-D	3.6	Malate dehydrogenase	Oxidoreductase	Ignicoccus islandicus DSM 13165	10	6.7
anti_CRISPR3672	SDK41378.1	AcrIC5	I-C	Pseudomonas delhiensis	60	4awh-C	4.4	Polymerase PA	Polymerase	Influenza A virus (A/California/04/2009(H1N1))	7	5
anti_CRISPR3673	WP_089394111.1	AcrIC5	I-C	Pseudomonas delhiensis	60	4awh-C	4.4	Polymerase PA	Polymerase	Influenza A virus (A/California/04/2009(H1N1))	7	5
anti_CRISPR3674	WP_080050315.1	AcrIC6	I-C	Pseudomonas sp. S-6-2	144	N/A	N/A	N/A		N/A	N/A	N/A
anti_CRISPR3675	WP_003294373.1	AcrIC7	I-C/I-E	Pseudomonas stutzeri	94	6c9i-G	5.3	DARP14 - Subunit B	Unknown	Pyrococcus horikoshii OT3, Pseudomonas aeruginosa PAO1	6	1.4
anti_CRISPR3676	EWC40192.1	AcrIC7	I-C/I-E	Pseudomonas stutzeri KOS6	94	6c9i-X	5.4	DARP14 - Subunit B	Unknown	Pyrococcus horikoshii OT3, Pseudomonas aeruginosa PAO1	6	1.4
anti_CRISPR3677	WP_074202337.1	AcrIC8	I-C/I-E	Pseudomonas aeruginosa	80	1mw8-X	6	DNA Topoisomerase I	Topoisomerase	Escherichia coli	8	2.6
anti_CRISPR3678	WP_101192668.1	AcrIE9	I-E	Pseudomonas pharmafabricae	75	4fwo-A	3.6	Propionate kinase	Kinase	Salmonella enterica subsp. enterica serovar Typhimurium str. LT2	4	3.8
anti_CRISPR3679	ETD02882.1	AcrIC9	I-C	Rhodobacter capsulatus DE442	79	3w21-B	4.4	Hypothetical protein	Unknown	Burkholderia ambifaria AMMD	13	6.8
anti_CRISPR3680	WP_017907426.1	AcrIC10	I-C	Xanthomonas sp. SHU 199	95	4je9-A	5.4	Soluble cytochrome b562	Transport protein	Escherichia coli	10	15.9
anti_CRISPR3681	WP_058195519.1	AcrIC10	I-C	Xanthomonas translucens strain CR31	94	2w6h-H	5.7	ATP Synthase Subunit Delta, Mitochondrial	Hydrolase	Bos taurus	16	13.7

anti_CRISPR3682	QEH00216.1	AcrIIA22	II-A	uncultured bacterium	54	7jta-A	11	NTF2-like nuclease/anti-CRISPR	Unknown	Clostridia bacterium	100	91.5

N/A in the column %IDSeq shows cases that did not pass the threshold in EMBOSS.

Table S5. Hardware specification for AlphaFold experiments

Name	Server 1
**CPU**	Xeon(R) Silver 4214R 2.40 GHz, 12core
**RAM**	32GB DDR4 Registered ECC PC4 2933 4 ea
**GPU**	GeForce RTX 3090, 24 GB, 1 ea
**SSD**	Segate FireCuda 530 Gen4 M.2, 4 TB
**HDD**	Western Digital, WD My Passport, 5TB

Table S6. Time to create a 3-D structure from one protein sequence (in seconds). We randomly picked five sequences and averaged the results. Since AlphaFold searches DB collation for generating representation, not only processor performance (GPU > CPU) but also storage input/output speed (SDD > HDD) greatly affects computation speed.

Processor	Storage	Generating Representation	Model 1	Model 2	Model 3	Model 4	Model 5	Total Time
CPU	HDD	3,009	1,408	1,301	1,251	1,250	1,153	9,372
GPU	HDD	3,599	170	130	93	100	93	4,185
GPU	SDD	1,191	245	125	85	89	84	1,820

Table S7. The best-fit-model of the phylogenetic tree of each Acr set using IQ-TREE ModelFinder.

Set	Number of Sequences	Best-fit Model	Criteria
**Set A**	109	LG+I+G4	BIC
**Set B**	98	LG+R4	BIC
**Set C**	236	LG+G4	BIC
**Set A+B**	207	LG+R5	BIC
**Set A+B+C**	443	LG+R5	BIC

LG: General matrix (Le and Gascuel, 2008)
I: allowing for a proportion of invariable sites
G4: discrete Gamma model (Yang, 1994)
R5: FreeRate model (Yang, 1994)
BIC: Bayesian Information Criterion (Schwarz, 1978)
